# Connecting the past and the future: Academic geographical (im)mobility of Chinese women academics throughout doctoral education

**DOI:** 10.3389/fpsyg.2022.987060

**Published:** 2022-12-05

**Authors:** Li Bao

**Affiliations:** ^1^School of Sociology and Population Studies, Nanjing University of Posts and Telecommunications, Nanjing, China; ^2^Faculty of Education and Social Work, University of Auckland, Auckland, New Zealand

**Keywords:** gender norms, women doctoral students, women academics, geographical mobility, a stylized repetition of acts

## Abstract

**Introduction:**

Academic geographical mobility is considered to be critical to academic excellence, but it is a gendered terrain. This study seeks to examine the career progression of Chinese women academics, as shaped by gender norms, regarding academic geographical (im)mobility throughout their doctoral education, in retrospect.

**Methods:**

To address this issue, driven by the Butlerian theoretical concept of “a stylized repetition of acts,” the present study analyzed the qualitative data from semi-structured interviews with seven Chinese women academics to investigate their academic geographical mobility decisions throughout their doctoral education based on contested discourses of traditional Chinese culture and the advantages of academic geographical mobility for their career advancement.

**Results and discussion:**

This study determined that, shaped by gender norms, stylized geographical academic (im)mobility for these Chinese female doctoral students operate in the condition of gender- norms maintenance to make them recognizable and understandable in social and institutional culture. However, it may have a negative impact on their future academic career progression.

## Introduction

In recent decades, the career progression of women academics, especially the factors hindering it, has drawn the attention of researchers. The gender gap between women and men in academics can be traced back to doctoral study. This study aimed to discuss the performance of Chinese women in academics, shaped by gender norms, with regard to academic geographical (im)mobility throughout their doctoral education, in retrospect. Through the interpretation of their experiences, this study explored how the performance of women academics is related to their experiences of academic geographical (im)mobility before their academic career begins. In this section, I examine the challenges that Chinese women academics and women doctoral students face, what academic geographical mobility might mean to individuals' academic career development, and how mobility can advance their academic careers in the context of contemporary Chinese higher education.

The Chinese higher education context is marked by gender norms, which have challenged women's career advancement in academia (Zhao and Jones, 2017). Chinese women are likely to show less ambition because of the different expectations of men and women in society (Larsson and Alvinius, 2020; Bao and Tian, 2022). Quantitative research shows that, compared to Chinese men academics, women's research productivity is lower for two main reasons: Chinese women academics are less likely to have sufficient academic networking to gain research resources and collaboration (Zhu and He, 2016; Bao and Tian, 2022); and Chinese women academics devote less time to research work mainly because of the imbalance of domestic housework allocation (Zhu and He, 2014).

To some extent, stereotypes and social expectations hinder the prospective academic development of Chinese female doctoral students (Zhou and Zhang, 2020). Sun and Zhang (2020) identified that Chinese female doctoral students face nonacademic gender pressure to balance work, study, and family. Chinese female doctoral students also report being less confident about future employment (Sun and Zhang, 2020); on average, they take longer to obtain their first work opportunity (Jin and Liu, 2011). After graduation, a disproportionately large number of women doctoral students choose domestic postdoctoral opportunities or job opportunities in familiar cities, which shows that women academics tend to choose less challenging but more stable work opportunities (Ma et al., 2014).

Researchers agree that academic geographical mobility is helpful for academic advancement (e.g., Ackers, 2004; Leung, 2017). Chinese universities aim to attract more academic returnees (Zhu and Wang, 2019)—those who study abroad for doctorates or have periodic overseas doctoral study and research experiences and then start their academic careers in a Chinese academy (Xu, 2009; Zhang and Yuan, 2014). The presumption is that returnees will have higher research productivity to help universities improve their academic development (Ye and Liang, 2019). Some Chinese universities claim overseas study experiences are preferred (Ye and Liang, 2019) and provide higher salaries, research funding, residence, and academic ranks to attract returnees (Pu, 2019). In Pu's (2019) interviews with 20 university presidents, increased research capacity, English language proficiency, and creativity were shown to be the advantages of returnees. Based on these advantageous policies and the developmental needs of Chinese higher education (Xinhua Press, 2021), overseas research experiences are more likely to help advance academic careers in Chinese universities. Being an academic returnee for Chinese women could contribute to their research capacity and make them more competitive in academia. Further, Chinese women and doctoral students may be more welcome in the academic job market if they have overseas research experiences.

This study aimed to investigate two research questions: (i) How do gender norms operate in doctoral studies in terms of geographical mobility? (ii) How do women academics connect their geographical mobility throughout their doctoral studies to their academic careers? In the next section, a review of the literature on gendered academic geographical mobility and Chinese traditional culture is presented.

## Gendered academic geographical mobility

Academic geographical mobility, described as “a gendered terrain” (Leung, 2017, p. 2711), in which more men than women academics participate, has been perceived worldwide as critical to academic excellence and career advancement. Though overseas research experiences are more likely to help women academics achieve prestige and recognition and is one of the key factors associated with academic career advancement (Yu and Wang, 2018), academic geographical mobility comes at a cost at the personal and family level (Suarez-Ortega and Risquez, 2014). Despite the potential advantages of becoming academic returnees, prior research demonstrated gender segregation in academic geographical mobility (Suarez-Ortega and Risquez, 2014; Bilecen and Mol, 2017; Yu and Wang, 2018). Leung (2017) argued that women are more likely to sacrifice their careers by leaving the profession or giving up opportunities when they are mobilizing for higher levels since it often causes tension with their partners (Acker and Armenti, 2004). For example, women academics in the USA and South Korea reported needing to sacrifice their mobility and research productivity for their families (Yoon and Kim, 2019). Women academics face personal and family difficulties associated with academic mobility, which makes the compatibility of these intersecting roles difficult (Suarez-Ortega and Risquez, 2014). Some women, as insiders, tend not to attribute the reasons to gender constraints, although outsiders may see this as a gender issue. Therefore, gender constraints, like religion and rituals, may sometimes work with a disguise. For example, Muslim women academics interviewed by Shah (2018) attributed the barrier to their academic geographical mobility to religious reasons rather than gender equality issues.

In Chinese universities, the participation of women academics in geographical mobility is restricted by traditional culture. In traditional Chinese culture, women are expected to behave as a “贤内助” (docile domestic helper) after they start families. Their husbands are usually considered the “breadwinners” with the responsibility and obligation to support their families, and women are encouraged to put them at the center of the family. According to Chinese cultural norms, a wife's principal duty is to ensure her husband's professional success (Cooke, 2005). Meanwhile, “从夫居” (patrilocal residence) is encouraged, which means Chinese women stay with their husbands' families after marriage (Fei, 2019, p. 104). Therefore, many Chinese women move to their husband's family homes to start a new life. Similarly, in contemporary academia, researchers found that the geographical mobility of women academics features *tied movers* (Ackers, 2004) and *linked lives* (Bailey and Mulder, 2017), suggesting that women academics are likely to move with their male partners when they face geographical mobility.

The focus of the present study is Chinese women academics' perception of academic geographical mobility when they were receiving doctoral education, based on contested discourses of the Chinese traditional culture, and the advantages academic geographical mobility may have for their career advancement. Prior research mainly focused on academic geographical mobility of international women academics (e.g., Suarez-Ortega and Risquez, 2014; Bilecen and Mol, 2017; Leung, 2017; Tam and Araújo, 2017). While there is limited domestic research connecting the lower productivity of women academics and their lack of overseas experience (e.g., Zhu and He, 2014), women academics' perceptions and contextualized reasons are usually understudied. This study aimed to explore academic geographical mobility in the Chinese higher education context and Chinese women's perceptions of academic performances in terms of their geographical mobility throughout the doctoral study. I conducted semi-structured interviews with seven Chinese women academics to address this issue, driven by the Butlerian theoretical concept of a stylized repetition of acts. The theory and the methods are introduced as follows.

## Theoretical framework: A stylized repetition of acts

For Butler (1988), gender is performed by “tacit collective performance” (p. 522). Gender norms are more likely to be implicit and contextualized in their operation. It may not be identified in social practice, but the result of the operation can be seen “clearly and dramatically in the effects” (Butler, 2004, p. 41). Butler (2006) called gendered appearances “naturalized knowledge, even though it is based on a series of cultural inferences, some of which are highly erroneous” (pp. xxiii–xxiv). The social practice of stylized gender features construct-specific gender norms embedded in an individual's behavior. Butler (2004) explains the following:

A norm is not the same as a rule, and it is not the same as a law. A norm operates within social practices as the implicit standard of normalization. Although a norm may be analytically separable from the practices in which it is embedded, it may also prove to be recalcitrant to any effort to decontextualize its operation. (p. 41)

In this operation of gender norms, Butler doubts the preexistence of gender subjects. She argued that, in a particular form of subjection to regulation, the gendered subject is produced.

How do gender norms work in social practice? Butler (1988) argued that “gender performances in non-theatrical contexts are governed by more clearly punitive and regulatory social conventions” (p. 527). When gender is produced and reproduced by subjects in culture, subjects must be subject to regulatory power to become subjects. In the resistance and violation of gender norms, regulatory power works “to identify those actions as inappropriate and problematic” (Lester, 2011, p. 145). Through this constant correction and adjustment of gender norms, subjects are regulated to form a homogenous performance, which is “the stylization of the body” (Butler, 1988, p. 519). This stylization is normalized as the regular and normative way of performing.

This study investigated and interpreted the narratives of the participants when they were women doctoral students using the Butlerian theoretical concept of “a stylized repetition of acts” (Butler, 2006, p. 191) through the operation of norms (Butler, 2001). Prior research showed that women academics, including those in China, are less likely to participate in academic geographical mobility (e.g., Suarez-Ortega and Risquez, 2014; Bilecen and Mol, 2017; Leung, 2017; Yu and Wang, 2018; Yoon and Kim, 2019). For the Chinese women academics in this study, the gender norms of academic geographical (im)mobility are operated in social and institutional discourses. Butler (2006) wrote that “Gender is an identity tenuously constituted in time, instituted in an exterior space through *a stylized repetition of acts*” (p. 191). In this study, the main theoretical argument is that, under the operation of gender norms in the Chinese context, women academics' participation in academic geographical mobility throughout the doctoral study is regarded as “a stylized repetition of acts.” This theoretical concept is helpful for this study because it may address the social and institutional factors that shaped the performances of Chinese women academics in relation to their academic geographical mobility during doctoral study. When they made decisions about mobility throughout the doctoral study, they were regulated by gender norms to avoid “inappropriate and problematic” actions (Lester, 2011, p. 145) to become subjects. Their repetition of these stylized acts may construct certain performances of women doctoral students, although their future academic careers might be disadvantaged by this repetition. For Butler (2006), “performativity is not a singular act but a repetition and a ritual, which achieves its effects through its naturalization in the context of a body, understood, in part, as a culturally sustained temporal duration” (p. xv). By constituting continuous repetition, women academics' coherent performances are naturalized. Hence, they produce “a stylized repetition of acts” (p. 191), which makes academic geographical mobility gendered.

## Data collection and analysis

The data for this study were extracted from a research program on the academic career growth of 20 Chinese women, which was approved by the Auckland Human Participants Ethics Committee (Reference Protocol Number: 024731). Half of them received domestic doctorates, while the others obtained doctorates overseas. All the participants, who gained doctorates before the interviews, were working in elite Chinese universities at the time of the interviews. These 20 participants were recruited through an online advertisement. All of them took part in this research voluntarily by responding to the advertisement. The relevant information in the formal consent form adequately informed the participants. The anonymity of participants was guaranteed before, during, and after the interviews. I stopped recruiting participants once the interview data were saturated, suggesting that a few new reports were found in the narratives. Semi-structured interviews were conducted mostly in Mandarin Chinese and then translated into English without the meaning intact. Both Chinese and English transcriptions were then sent to the participants for any possible corrections and further clarifications before use. Because of the pandemic, all the interviews were undertaken through online video or audio meetings; they took 45–180 min. The duration varied because the interviews were conducted based on feminist storytelling to give the participants ample space to be more powerful while reorganizing their life stories. Hence, I did not set a time limit during the restoration of their life stories (Bochner and Riggs, 2014).

The participants were asked open-ended questions, such as “Why did you choose to do your doctorate overseas/domestically?” and “Can you please tell me about the difficulty of your doctoral study?” All the participants were encouraged to recall their experiences throughout doctoral education and draw connections with their current academic careers and personal lives. During the interviews, participants' narratives were reconstructed, reproduced, and “always undergoing revision” (Butler, 2001, p. 27) as a means of the performative act. When the participants recalled their experiences, they were in the reflexive process of reconstructing and reproducing their performative acts. All 20 participants were asked about geographical (im)mobility throughout their doctoral education. I selected seven participants who provided adequate data on the experiences constructed by gender norms according to their narratives (see Table 1). After the interviews, the participants were allowed to read and edit their transcripts to double-check for accuracy. All the participants consented to the transcripts.

**Table 1:** Background of the participants.

**Pseudonym**	**Discipline**	**Years after gaining a doctorate**	**Country/district gaining doctorates**
An	Sociology	8	Mainland China
Bai	Ethics	2	Belgium
Danni	Literature	2	Mainland China
Luna	Sociology	2	Hong Kong SAR
Naya	Education	10	Mainland China
Rui	Education	3	New Zealand
Zhong	Linguistics	7	Mainland China

In this study, I analyzed the interview data in the following three steps: First, I divided the transcription into different themes, such as immobility, before the doctoral study. These themes were shared and checked in a writing group meeting organized by the faculty of education and social work at the University of Auckland. Second, I revisited the data and chose the quotes for analysis driven by the Butlerian theory of “a stylized repetition of acts.” Third, I contextualized these data in the context of Chinese society and higher education for further analysis.

## Findings: Geographical (im)mobility throughout doctoral education

The research findings are presented chronologically throughout doctoral education to show how the participants perceived their academic geographical (im)mobility at the intersection of contested discourses.

### “I considered all the factors”: Immobility before doctoral education

A few participants in this study reported that they had struggled with overseas and international doctoral study opportunities when they were seeking them. Naya, who received a domestic doctorate, considered overseas doctoral education in the hope of “*better development*” in her future academic career. However, her decision-making was influenced by her male partner. She said the following:

*I needed to consider the length. In the second year of my master's study, my supervisor asked me if I wanted to continue my doctoral study under his supervision. I thought since I've got the “easy mode,” why take the “hard” one?... If I went to the US, it might have taken me five or more years to graduate, but I finished my doctoral study here in 3 years. And my boyfriend and I can't keep long-distance for years. Therefore, I considered all the factors*.

In this situation, pursuing a doctorate away from her boyfriend might have sabotaged Naya's relationship. Despite her desire to study abroad, she was willing to fulfill the social expectations of women during her doctoral study. After all, in Chinese social discourse, women doctoral students are at the “best ages for marriage and childbirth” (Gui, 2017). Even though the international mobility of doctoral students improves the quality of doctoral training and enhances the academic development of early-career researchers (Leyman, 2009), when the personal pursuit of academic development threatened to shake the relationship with her significant other, Naya chose to secure her relationship to meet the “implicit standard of normalization” (Butler, 2004, p. 41) of being a woman.

Similarly, Zhong dreamed of studying abroad for her doctorate, especially after being a 1-year visiting scholar in the US. However, she eventually chose to continue with her doctoral study at the university she was working at because “*if I had my doctoral education in the US or Canada, I would have little income during doctoral study. It was unacceptable because I have to raise my daughter.”* The contested familial responsibility constrained the possibilities for Zhong in her doctoral education. She had to consider the financial implications of supporting herself if she studied overseas. Despite her strong motivation to receive overseas doctoral education, she eventually chose to continue with her doctoral study at the local university where she worked, not only for the salary but also because “*My ex-husband would not let me visit my daughter if I went abroad. I can't leave her in China*.” For Zhong, being a doctoral student overseas was a huge commitment. It would require family sacrifice and might create child custody issues, although it might likely benefit her career development. It was also unlikely to satisfy the expectations of gender norms as a mother because she could not physically take care of her daughter anymore if she went abroad. Her agency to make a difference in academic work was strong. However, after careful consideration, she still prioritized family responsibilities under the regulation of gender norms to avoid the consequences of challenging social conventions (Butler, 1988).

Naya and Zhong did not change their plans to seek doctorates. However, the “*all the factors*” Naya considered, and Zhong's choice between doctoral education and caring responsibilities, indicated the decision of how and where to do a doctorate was not entirely up to them. Naya concluded that her choice was “*the most efficient one*,” while Zhong thought it was the “*rational choice*.” However, they were both aware that doing doctorates overseas may have contributed more to their academic careers. They were both working on work-life balance before they entered academia to dodge the social consequences of going against gender norms. In other words, both Naya and Zhong compromised their career aspirations to perform their gender roles. With overseas research experiences, women academics are more likely to earn prestige and recognition in academia, partially overcoming the social disadvantage of gender, which demands that women academics make adjustments required by cultural and gender norms (Tam and Araújo, 2017). Therefore, Naya and Zhong presented “the stylization of the body” (Butler, 1988, p. 519) to form a homogenous performance in relation to their academic geographical immobility. They knew that, shaped by the contested norms, self-sacrifice for them was inevitable. In their academic career development, Naya and Zhong both admitted that their paths may have been different if they had done doctorates abroad. They also sought other ways to compensate for the overseas doctoral education they did not receive: During Naya's doctoral study, she spent 1 year studying in the US as a visiting doctoral student; after doctoral graduation, Zhong reached out to a doctoral supervisor working in a leading university and learned from this supervisor about English academic writing for more than 5 years. In Chinese culture, because of the culture of “从夫居” (patrilocal residence), social discourse encourages women to stay or move with their husbands. Therefore, Chinese women are less likely to be mobile as individuals, even if it benefits their careers. However, because social discourse emphasizes men's career success, relationship maintenance and family responsibilities are less likely to interfere with their mobility. The interview data in the next sections also support this.

### “To be honest, I am very traditional”: Transnational mobility for doctoral education

In Bai's case, what hindered Naya's path to overseas doctoral study was her catalyst. She described her male partner's company and encouragement:

*Another important reason was that my husband [her boyfriend at the time] had been planning to study abroad for years... he influenced me. The first time he said we should apply for overseas doctorates together, I thought it was incredible; I had never thought about this option... To be honest, I am very traditional... Though I was going to pursue a Ph.D., if the big issue [marriage] in my life hadn't been tackled, I would have been a little scared. I am easily influenced [by other people] to care about what others are thinking [about me]*.

Before the start of her doctoral study, Bai's concerns were regarding her possible failure to fulfill the social expectation of women, which is to be married or have a serious long-term relationship at the “marriageable” age (Gui, 2017, p. 1924). Bai was scared about her potential violation of social discourse because she is “*very traditional*.” For women, starting a family is “an indispensable and highly time-sensitive step in every individual's life trajectory” (Gui, 2017, p. 1936). Bai's worry about how others saw her reflected the power of gender norms. Bai added, “*Many of my old friends didn't pursue doctorates, and they got married and had kids [when I was doing my doctorate]*.” Thus, she felt she needed to fulfill gender expectations to perform a “tacit collective agreement” (Butler, 1988, p. 522) by following her friends' life trajectories. The overseas doctoral education she took was built on this presumption of *proper* repetition of gender performances.

Rui, who acquired her doctorate overseas, echoed Bai: “*I want to thank my husband for coming to this country with me. I would not have chosen to study overseas if he had not been supportive*.” Gendered strategy in transnational mobility is often linked with family and parenthood (Nikunen and Lempiäinen, 2020). With her husband and daughter's company, Rui completed her doctoral study and continued with a post-doctoral research fellowship at the same university. These overseas experiences largely advanced her academic career after she returned to China.

These two women academics' decisions to study abroad for doctorates were shaped by their “goal of self-realization” (Ye, 2018, p. 226). Being academic returnees, they both claimed that the experience of doing overseas doctorates was “*very helpful*” (Bai) and “*beneficial*” (Rui) to their future career development. It shows that women are capable of achieving academic excellence if they have the opportunity to exercise geographical mobility. Tied movers (Ackers, 2004) may typically allude to passive followers, typically women whose partners relocate for career progress (Clerge et al., 2017). However, in both of these scenarios, being tied to movers also affords women the chance to further their education. However, this means of mobility reduces women's autonomy by maintaining their affiliated social status. If their career advancement hinders the mobility of their male partners, women may sacrifice their careers.

### “Girls didn't make the choice”: Choices of (im)mobility after graduation

An explanation of her decision after doctoral graduation:

*An: I think if I had listened to my boss and done an overseas postdoc, I could've been better [in my academic career development]*.

*Interviewer: Do you think it may relate to your female identity?*

*An: Yes. [In real life,] If I go abroad, we [An and her boyfriend] had to be [in a] long-distance [relationship]. It was inconvenient...*.

*Interviewer: You said many of your male colleagues took the overseas post-doc. Is it because of their research capacity [being better than that of their female counterparts], or were those just their choices?*

*An: Actually, everyone can have this opportunity, but girls didn't make a choice*.

An was considering her intimate relationship in the transition between a doctoral study and academic career, which was similar to the performances of Naya and Bai before their doctoral study. According to An's narrative, in the top domestic university she studied at, women doctoral graduates were less likely than men to choose overseas postdoctoral positions, and they regarded men taking these positions as the norm, even when they were academically capable of receiving equivalent overseas postdoctoral appointments. An and her female peers were repeating their senior women doctoral colleagues' pursuit of domestic post-doctoral positions or domestic academic working positions. In this case, the gender norms were maintained and enhanced by the homogeneous performance of women doctoral students at An's university.

Post-doctoral appointments contribute to early career development (Webber and Canche, 2018). The choices after doctoral graduation, usually between taking domestic post-doctoral appointments and accepting academic positions, are more likely to constrain women doctoral graduates' research capacity development than their male counterparts who take overseas post-doctoral appointments. Identifying with gender norms, An and her female peers categorized themselves differently from men. They leaned toward different decisions, even though they were aware it might hinder their career development if they made “*a sacrifice*.” These women doctoral students showed their conformity and loyalty to gender norms by *making sacrifices or compromises* for their future careers. Through their repeated performative acts shaped by gender norms, the women doctoral students tended to or had to choose conservative career development paths, which may have started to widen the gender gap before their academic careers. After all, did the participants know what their decisions might lead to? An provided her explanation:

*Sometimes, we [me and my female doctoral colleagues] realized it [dropping the opportunities for overseas postdocs] was a sacrifice, but this thought made us unhappy. We cannot think of it [in this way]. Yet every step, every choice, determines future [career] development. These are all turning points*.

Academic geographical mobility is closely tied to gender inequality (Bilecen and Mol, 2017). An and her women doctoral colleagues understood the possible consequences of their decisions; nevertheless, they embarked on this path. Her expression “*we cannot think of it*” shows “a loyalty to some bond in the present or the past,” which is “simultaneously preservation as well” (Butler, 2006, p. 67). An and her peers put on the “mask” to “conceal this loss” of advanced academic careers; however, in this way, this loss is maintained “through its concealment” (Butler, 2006, p. 67). They performed this stylized repetition of gender norms by making potentially inferior decisions.

The domination of social discourse after graduation emphasizes the performances of gender norms by women doctoral students. Looking back at her decision about overseas post-doctoral positions, An explained, “*If I were a man, if I didn't have a partner who cared about me, I might have made a different choice.”* An's narrative showed the different gender norms of women and men in career development. Her stylized repetition of actions reflected her concerns about social consequences. Danni's story of her doctoral colleagues resonated with An's narrative:

*When some of my female doctoral colleagues were looking for academic positions, they accommodated themselves to the working locations of their husbands. Therefore, they went to some platforms in lower level [universities], compared to their academic capacity. It makes their future academic career development harder. I am not very optimistic about them*.

Danni gave the examples of her doctoral peers to show the relationship of their choices to future academic career development. She believed that if these female doctoral students started their academic careers in higher-ranked universities, their academic performances would be better. She also described the regret of her doctoral supervisor, “*She [one of Danni's female doctoral colleagues] was very smart in doctoral study. How can she stop doing research after having two children?*”

This had an adverse effect on the future career development of some female doctoral students, something that was also noticed by Luna's doctoral supervisor. Luna claimed:

*In recent years, men doctoral supervisors in my department have accepted fewer women doctoral students because some of our women doctoral graduates did not continue their academic careers after becoming academics. They concentrated on their families. It made male doctoral supervisors feel that their effort to train these female doctoral students was wasted. Therefore, in my Ph.D. study, our department's proportion of new male doctoral students was increasingly larger*.

Gender norms guide female doctoral students in different directions, which may reduce their agency to be ambitious and accomplished in their academic careers. More importantly, this tendency is reinforced by supervisors, the representatives of authority with power over doctoral students. When women doctoral students' disadvantaged (im)mobility became “a ritual” and “a culturally sustained temporal duration” (Butler, 2006, p. xv) in An and Danni's narratives, doctoral supervisors reacted negatively to the attrition of women doctoral students in their future academic careers. As En said, in academia, the discrimination against women is “*implicit*” and “*unidentifiable*,” and “*when the policymakers are making decisions, you can't tell if it relates to gender*.” In Luna's narrative, her supervisor found that female doctoral students were more likely to underperform than their male counterparts after graduation. Therefore, her male supervisor explicitly truncated the opportunities for women to receive doctoral education by limiting enrollment. For Luna, this gender discrimination against women doctoral students was explicit and identifiable. However, as En said, this exclusion was hard for women applicants to identify because they may have attributed their failure to other reasons, such as unsatisfactory performances in the interviews.

## Discussion: Past mobility and future academic career

In retrospect, the participants reported how gender norms shaped their performances in academic geographical (im)mobility throughout their doctoral education. Here, I discuss the findings from two aspects of academic geographical (im)mobility: what it means to be a woman and the connection with their academic career development.

### (Im)Mobility for being women

From the narratives, when these women academics recalled their experiences of academic geographical (im)mobility throughout doctoral education, they considered gender norms for the male-dominated culture they were in. They reported two ways of (im)mobility: moving with their husbands and staying with their families.

For one, some of the women academics chose to move with their male partners when deciding about academic geographical mobility throughout their doctoral education. This mobility was usually based on the great benefits of the husbands' career development, consequently limiting women's academic choices. For example, Bai completed her doctoral study in Belgium because her male partner got a Ph.D. admission in a nearby European country. Butler (2006) claimed that agencies perform functions under regulation. When Bai went abroad with her male partner for doctoral study, her agency operated within the framework of gender norms. Encouraged by Chinese culture, she conformed to the gender norm of having a potential marriage at an early age by moving with her fiancé. Her agency of seeking a doctorate operates under the regulation of being recognized as a woman.

For another woman, if the women academics suppressed their desire to seek overseas doctorates or research experiences, it was due to their desire to stay for their families. Some participants (e.g., Naya, Zhong, and An) truncated their agency by giving up the opportunities of receiving overseas doctoral education or taking post-doctoral positions, which limited their academic geographical mobility and thus might have prevented them from having a stronger start in their academic careers. Moreover, according to the narratives by An and En, women tended to have fewer opportunities to gain positions in doctoral study and academia. Their experiences of the interaction between the agency of academic development and gender norms constructed the tension in those women's academic careers and then shaped their performance in their academic careers. Notably, even if some participants gave up or were excluded from some academic opportunities, none of them showed explicit resistance; they thought they were making rational choices when they reported these experiences as repeated performative acts in the interviews. Therefore, I argue that shaped by gender norms, geographical academic (im)mobility for these Chinese female doctoral students is “instituted in an exterior space through a stylized repetition of acts” (Butler, 2006, p. 191): (im)mobility in the condition of gender norms maintenance. This stylized mobility, described as “naturalized knowledge” (Butler, 2006, p. xxiii), which may pose problems for their autonomy and career progression, is supported by Chinese social discourse, which expects women to prioritize their families.

### Connection with their academic career development

In the operation of power relations to maintain gender norms, the participants were subject to those norms by forming stylized repetitions of acts throughout their doctoral education. This agreement makes women “recognizable and understandable” (Butler, 2001, p. 26) and contests their singularity. Moreover, it is likely to disadvantage women in academia. On this basis, in the interviews, Naya asked, “*Where are the female doctoral students after graduation?*” Danni proposed a similar inquiry in detail:

*Where are the female colleagues now? When I was doing my master's degree and Ph.D., there were a lot of female students; it is not a male-dominated discipline. Where are they? When I was at an academic conference of fifty people, the proportion of women in academia was generally between one-fourth and one-third. However, in my doctoral study, the proportion of female students was higher than half*.

Naya and Danni were concerned about the disproportionate participation and presentation of women academics at academic events and in academia. In Danni's observation, this shift from female-dominated classrooms to male-dominated workplaces may be caused by the disadvantages they experienced in their academic careers. Based on the findings, the lack of advantageous academic geographical mobility throughout doctoral education may reduce the competitiveness of women in academia, and their repeated performances are likely to reproduce gender norms.

On the one hand, as Butler (2006) writes, “construction is not opposed to agency; it is the necessary scene of agency, the very terms in which agency is articulated and becomes culturally intelligible” (p. 201). This lack of academic geographical mobility throughout the doctoral study shaped, to some extent, the career development of these women academics. Meanwhile, the need for cultural acceptability limited their freedom of choice. For example, the fact that she and her female doctoral colleagues did not apply for overseas post-doctoral opportunities did not stop their pursuit of academic advancement. Nevertheless, their decisions to give up potential career advantages made them conform to gendered societal norms, which is more likely to encourage men's career ambitions.

On the other hand, throughout doctoral education, women doctoral students were confronted with the expectation of normative women's performance, which is already being shaped by gender norms. For Butler (2001), “I become recognizable through the operation of norms” (p. 25). The participants were subject to the regulation of gender norms to make them recognizable (Butler, 2001, 2004). Their academic careers were also reformed for this gendered social expectation, along with the performances of gender norms right through to doctoral graduation. For example, Naya, Bai, and An would prefer to start a family before a certain age, even though their academic career aspirations might contest this goal. This stylized repetition of acts, “based on a series of cultural inferences” (Butler, 2006, p. xxiv), forms female doctoral students' decisions regarding academic geographical mobility. It is likely that, as mentioned, “*future career development is determined, or limited*,” which may answer Danni's question: “*Where are the women academics now?*”.

## Conclusion

In this study, I have explained the participants' performances of gender norms throughout their doctoral education by qualitatively examining how academic geographical (im)mobility is shaped by gender norms throughout their doctoral study and how the stylized repetition of acts is formed to affect their current academic performances. When women academics made decisions before and after doctoral study, they were more likely to prioritize fulfilling social expectations to become *normative* women. Their stylized choices and decisions conformed to societal gender norms but interfered with the career trajectories of these women academics before they entered academia. However, notably, these norms of mobility are “not as fixed as we generally assume [them] to be” (Butler, 2006, p. xv) if women doctoral students repeatedly perform with the agency of academic excellence in making decisions about geographical mobility, which may lead to the subversion of gender norms and gradually change the social and institutional discourses.

Although this study merely focused on a small number of participants, the analysis, based on semi-structured interviews, has provided knowledge of how gender norms shape women's academic geographical mobility throughout doctoral education by connecting doctoral study and academic careers and may explain some women academics' disadvantages in career development. This study shows that gender issues in doctoral studies may regulate women doctoral students' choices and thus affect their future academic careers. Therefore, the findings require universities, supervisors, and educational administrators of graduate students, especially Chinese female doctoral students, to provide study guidance and career suggestions based on their research capacity instead of their gender. This study also aimed to raise women doctoral students' awareness of gender norms when deciding their future careers.

The limitations of this study reflect two aspects. For one, the disciplines of the participants are mainly social sciences. Geographical mobility for academics in scientific research may affect their career progression differently. For another, geographical mobility does influence academic careers to some extent; however, there are more complex reasons for career progression. Further studies may consider taking women academics in STEM fields as participants. Intersected factors that affect geographical mobility and academic career advancement deserve further in-depth study. Gender studies may conduct interviews between groups of men and women academics for comparison.

## Data availability statement

The raw data supporting the conclusions of this article will be made available by the authors, without undue reservation.

## Ethics statement

The studies involving human participants were reviewed and approved by, Granting body: University of Auckland Human Participants Ethics Committee Reference Protocol Number: 024731. Title of study: Chinese women academics' career development. The patients/participants provided their written informed consent to participate in this study.

## Author contributions

LB contributed to the conception and design of the study, conducted the data analysis, wrote the manuscript, organized the database, and validated the data analysis.

## Funding

The research leading to these results has received funding from the Ministry of education of Humanities and Social Science project (Grant number 22YJC840001), and the Project of Social Science Foundation of Jiangsu Province (Grant number 22JYB024).

## Conflict of interest

The author declares that the research was conducted in the absence of any commercial or financial relationships that could be construed as a potential conflict of interest.

## Publisher's note

All claims expressed in this article are solely those of the authors and do not necessarily represent those of their affiliated organizations, or those of the publisher, the editors and the reviewers. Any product that may be evaluated in this article, or claim that may be made by its manufacturer, is not guaranteed or endorsed by the publisher.
